# Association of Crohn’s disease-related chromosome 1q32 with ankylosing spondylitis is independent of bowel symptoms and faecal calprotectin

**DOI:** 10.7717/peerj.5088

**Published:** 2018-06-26

**Authors:** Rebecca L. Roberts, Mary C. Wallace, Andrew A. Harrison, Douglas White, Nicola Dalbeth, Lisa K. Stamp, Daniel Ching, John Highton, Tony R. Merriman, Philip C. Robinson, Matthew A. Brown, Simon M. Stebbings

**Affiliations:** 1 Department of Surgical Sciences, Dunedin School of Medicine, University of Otago, Dunedin, New Zealand; 2 Department of Medicine, University of Otago Wellington, Wellington, New Zealand; 3 Department of Rheumatology, Waikato Clinical School, Waikato Hospital, Hamilton, New Zealand; 4 Department of Medicine, University of Auckland, Auckland, New Zealand; 5 Department of Medicine, University of Otago Christchurch, Christchurch, New Zealand; 6 Department of Rheumatology, Timaru Hospital, Timaru, New Zealand; 7 Department of Medicine, Dunedin School of Medicine, University of Otago, Dunedin, New Zealand; 8 Department of Biochemistry, University of Otago, Dunedin, New Zealand; 9 School of Medicine, Faculty of Medicine and Biomedical Sciences, University of Queensland, Brisbane, QLD, Australia; 10 Institute of Health and Biomedical Innovation, Queensland University of Technology, Translational Research Institute, Princess Alexandra Hospital, QLD, Australia

**Keywords:** Dudley Inflammatory Bowel Symptom Questionnaire, Axial inflammation, Crohn’s disease, Kinesin Family Member 21B, Bowel inflammation

## Abstract

**Background:**

Genome-wide association studies have identified a plethora of risk genes for both Crohn’s disease (CD) and ankylosing spondylitis (AS). A subset of genes found to be risk factors for CD have also been found to be risk factors for AS. The objective of our study was to assess whether CD risk genes were associated with non-invasive clinical markers of gut inflammation in patients with AS, indicating a potential subset of patients with clinical as well as genetic overlap.

**Methods:**

A total of 308 Caucasian patients who fulfilled the modified New York Criteria for AS, were assessed for bowel symptoms using the Dudley Inflammatory Bowel Symptom Questionnaire (DISQ). Of these patients, 157 also had faecal calprotectin measured. All AS patients and 568 healthy controls were genotyped for 10 CD risk loci using predesigned single nucleotide polymorphism (SNP) genotyping assays. Chi-square analysis was used to test for association between genotype and DISQ score and faecal calprotectin level.

**Results:**

The minor allele of two SNPs, one in chromosome region 1q32 SNP (rs11584383), and one in the gene coding for *IL23R* (rs11209026) conferred protection against AS. Only the association of 1q32 remained significant after Bonferroni correction for multiple testing. Stratification by DISQ score and faecal calprotectin did not influence the association of 1q32 with AS.

**Conclusion:**

In patients with AS, the association of the CD 1q32 SNP was independent of non-invasive markers of bowel inflammation. Other CD related SNPs were not found have a significant association with AS.

## Introduction

In recent years a new paradigm has developed around ankylosing spondylitis (AS) in which AS is considered part of a spectrum of spondyloarthropathies that also includes Crohn’s disease (CD) (Raychaudhuri & Deodhar, 2014). These two conditions not only demonstrate many common phenotypic characteristics, but also share a common genetic risk. Furthermore, gut inflammation has been identified a frequent sub-clinical manifestation of AS (Mielants et al., 1988, 1991, 1995a, 1995b). Around 10% of AS patients go on to develop true CD, and a similar proportion of CD patients develop AS. Subclinical terminal ileitis is present in around 60% of patients with AS and 10–50% of patients with CD have radiographic evidence of sacroiliitis (Mielants et al., 1988, 1991, 1995a, 1995b; Stebbings et al., 2012).

The Icelandic genealogy database has demonstrated the coinheritance of CD and AS (Thjodleifsson et al., 2007). Not only do first and second degree relatives of a patient with CD have a heightened risk of developing CD themselves, but they are also at increased risk of developing AS, compared to the general population (Thjodleifsson et al., 2007). The reciprocal relationship is seen in families with AS. More recently genome-wide association studies (GWAS) have identified a plethora of risk genes for both conditions (Australo-Anglo-American Spondyloarthritis et al., 2010; Ellinghaus et al., 2016; The Australo-Anglo-American Spondyloarthritis Consortium (TASC) et al., 2011; Jostins et al., 2012). A subsequent meta-analysis of three CD GWAS identified further CD risk genes (Barrett et al., 2008). Danoy et al. (2010) took the 53 risk loci identified by this meta-analysis to be most strongly associated with CD, and genotyped them in a cohort of 2,773 patients with AS and 2,215 controls of Caucasian ancestry. Of these loci, eight were also found to associate strongly with AS (Danoy et al., 2010), indicating these loci are risk factors not only for CD, but also for AS.

Non-invasive markers of bowel inflammation have been piloted in several observational studies in AS. The Dudley Inflammatory Bowel Symptom Questionnaire (DISQ) (Stebbings et al., 2012) has been shown to identify patients with spondyloarthritis who have a high burden of bowel symptoms. Faecal calprotectin is widely used to identify patients with active bowel inflammation in CD and has been shown to be elevated in patients with AS who have histological evidence of bowel inflammation (Cypers et al., 2016).

We hypothesized that shared risk loci may influence the clinical spectrum of bowel inflammation in AS, and hence may associate with an excess of adverse bowel symptoms in patients who have AS but no evidence of overt CD. In order to investigate this hypothesis, the objective of our study was to assess whether the eight CD risk loci most strongly associated with AS were also associated with non-invasive indicators of bowel inflammation in patients with AS.

## Materials and Methods

### Study participants

A total of 308 Caucasian patients, who fulfilled the modified New York criteria for AS and who had no history of inflammatory bowel disease (IBD), were recruited from centres in New Zealand and Australia. A total of 568 New Zealand Caucasian healthy individuals aged over 17 years, with no personal or family history of spondyloarthritis or IBD, were used as controls (Simkins et al., 2005). Informed written consent was obtained from all study participants, and ethical approval for this study was granted by the Upper South and Lower South Regional Ethics Committees of New Zealand and the Princess Alexandra Hospital Research Ethics Committee, Brisbane, Australia (Ethics approval ref: MEC/09/08/084).

### Non-invasive markers of bowel inflammation in patients with AS

Bowel symptoms were evaluated quantitatively in all patients with AS using the spondyloarthritis modification of the DISQ, a validated outcome measure comprising 15 questions assessing bowel symptoms in axial spondyloarthritis (Stebbings et al., 2012). Each question was scored on a five-point numerical rating scale (from 0 = none/never to 4 = incapacitating). A DISQ score of ≥11 indicated the presence of bowel symptoms sufficient to affect quality of life (Stebbings et al., 2012). Faecal calprotectin was used as an objective marker of bowel inflammation, and was measured in a subset of patients (*n* = 157) using ELISA (Reference range: <50 mg/kg) (Canterbury Health Laboratories, Christchurch, New Zealand).

### DNA extraction and genotyping of confirmed CD risk loci

Guanidium isothiocyanate extraction was used to collect DNA from all study participants. Each participant was then genotyped for the confirmed CD risk loci (Danoy et al., 2010), 1q32 (rs11584383), *JAK2* (rs10758669), *CDKAL1* (rs6908425), *IL12B* (rs100454310), *ZPBP2* (rs2872507), *MUC19/LRRK2* (rs11175593), *STAT3* (rs744166), *IL23R* (rs1343151, rs10489630, rs11209026). Genotyping was performed with pre-designed TaqMan^®^ single nucleotide polymorphism (SNP) genotyping assays as per the manufacturer’s instructions using a Roche LightCycler 480 Real-time PCR system. End-point genotyping analysis software was used to assign genotypes to each study participant. A total of 10% of samples were re-genotyped to determine the accuracy of each TaqMan^®^ assay. All assays were found to exhibit 100% agreement between original and repeat genotype calls.

### Statistical analyses

The software program PLINK was used both to determine deviations from HWE, and to conduct chi-square tests to detect possible associations of genotype with AS and DISQ score. Associations were considered significant if, after Bonferroni correction (0.05/10), *p* < 0.005.

## Results

In our dataset, the minor alleles of 1q32 SNP rs11584383 (*p* = 0.0006, OR = 0.68, 95% CI [0.53–0.84]), and *IL23R* SNP rs11209026 (*p* = 0.02, OR = 0.57, 95% CI [0.35–0.93]) both conferred protection against AS (Table 1). However, only the association of 1q32 SNP rs11584383 remained significant after Bonferroni correction for multiple testing (*p* = 0.006). With stratification by DISQ score, the association of 1q32 SNP rs11584383 remained significant in both AS patients who had minimal bowel symptoms (*p* = 0.004) and those with clinically significant bowel symptoms (*p* = 0.02) (Table 2). Whilst stratification by faecal calprotectin showed an apparent difference between genotype groups (Table 2), comparison of ORs demonstrated this difference was not significant.

**Table 1:** Allele and genotype frequencies of CD risk loci in patients with AS and controls (ctls).

Locus/gene	SNP	Phenotype	Genotype			MAF	*p*	OR [95% CI]
*JAK2*	rs10758669		**A/A**	**A/C**	**C/C**	**C**		
		AS	124 (0.40)	138 (0.45)	46 (0.15)	230 (0.37)	0.32	1.11 [0.90–1.37]
		Ctl	208 (0.42)	234 (0.47)	57 (0.11)	348 (0.35)		
1q32	rs11584383		**T/T**	**T/C**	**C/C**	**C**		
		AS	180 (0.60)	104 (0.35)	16 (0.05)	136 (0.23)	6 × 10^−4^	0.68 [0.53–0.84]
		Ctl	247 (0.48)	226 (0.44)	45 (0.09)	316 (0.31)		
*CDKAL1*	rs6908425		**C/C**	**C/T**	**T/T**	**T**		
		AS	204 (0.66)	87 (0.28)	17 (0.06)	121 (0.20)	0.90	1.02 [0.80–1.30]
		Ctl	342 (0.65)	165 (0.31)	23 (0.04)	211 (0.20)		
*IL12B*	rs10045431		**C/C**	**C/A**	**A/A**	**A**		
		AS	152 (0.51)	128 (0.45)	19 (0.06)	166 (0.28)	0.65	0.95 [0.76–1.19]
		Ctl	254 (0.50)	211 (0.42)	40 (0.08)	291 (0.29)		
*ZPBP2*	rs2872507		**G/G**	**G/A**	**A/A**	**A**		
		AS	76 (0.25)	140 (0.46)	87 (0.29)	314 (0.52)	0.03	1.25 [1.02–1.53]
		Ctl	145 (0.29)	248 (0.50)	108 (0.22)	464 (0.46)		
*MUC19_LRRK2*	rs11175593		**C/C**	**C/T**	**T/T**	**T**		
		AS	290 (0.97)	9 (0.03)	0 (0.00)	9 (0.02)	0.70	1.18 [0.51–2.75]
		Ctl	536 (0.98)	12 (0.02)	1 (0.00)	14 (0.01)		
*STAT3*	rs744166		**T/T**	**T/C**	**C/C**	**C**		
		AS	99 (0.33)	151 (0.50)	55 (0.18)	261 (0.43)	0.77	1.03 [0.84–1.26]
		Ctl	181 (0.35)	243 (0.47)	98 (0.19)	439 (0.42)		
*IL23R*	rs1343151		**C/C**	**C/T**	**T/T**	**T**		
		AS	129 (0.53)	85 (0.35)	31 (0.13)	147 (0.30)	0.06	0.80 [0.64–1.01]
		Ctl	233 (0.42)	257 (0.46)	65 (0.12)	387 (0.35)		
*IL23R*	rs10489630		**T/T**	**T/G**	**G/G**	**G**		
		AS	106 (0.34)	143 (0.46)	59 (0.19)	261 (0.42)	0.57	0.94 [0.77–1.15]
		Ctl	160 (0.31)	269 (0.51)	95 (0.18)	459 (0.44)		
*IL23R*	rs11209026		**G/G**	**G/A**	**A/A**	**A**		
		AS	286 (0.93)	20 (0.07)	1 (0.00)	22 (0.04)	0.02	0.57 [0.35–0.93]
		Ctl	497 (0.88)	65 (0.12)	2 (0.00)	69 (0.06)		

**Note:**

SNP, single nucleotide polymorphism; MAF, minor allele frequency; *p*, unadjusted allelic *p*-value; OR [95% CI], odds ratio and 95% confidence intervals.

**Table 2:** Allele and genotype frequencies of 1q32 SNP rs11584383 in patients with AS and controls (ctls) stratified according to DISQ score and calprotectin level.

Phenotype	1q32 genotype			MAF	*p*	OR [95% CI]	*P*_difference_^Φ^
DISQ	T/T	T/C	C/C	C			
AS (≤10)	107 (0.60)	65 (0.36)	8 (0.04)	81 (0.23)	0.004	0.66 [0.50–0.88]	
AS (≥11)	73 (0.61)	39 (0.33)	8 (0.07)	55 (0.23)	0.020	0.68 [0.49–0.95]	0.893
Ctl	247 (0.48)	226 (0.44)	45 (0.09)	316 (0.31)			

Phenotype	1q32 genotype			MAF	*p*	OR [95% CI]	*P*_difference_^Ψ^
Calprotectin	T/T	T/C	C/C	C			
AS (0–149)	75 (0.65)	37 (0.32)	4 (0.04)	45 (0.20)	0.00071	0.55 [0.39–0.78]	
AS (≥150)	23 (0.56)	14 (0.34)	4 (0.10)	22 (0.27)	0.486	0.84 [0.50–1.39]	0.179
Ctl	247 (0.48)	226(0.44)	45 (0.09)	316 (0.31)			

**Notes:**

DISQ, Dudley Inflammatory Bowel Symptom Questionnaire; MAF, minor allele frequency; *p*, unadjusted allelic *p*-value; OR [95% CI], Odds ratio and 95% confidence intervals.

Φ *P*_difference_ between AS patients with DISQ ≤10 and ≥11.

Ψ *P*_difference_ between AS patients with calprotectin 0–149 and ≥150. A faecal calprotectin of ≥150 g/kg was considered to represent definite bowel inflammation.

## Discussion

It is well recognized that many AS patients experience clinically significant bowel symptoms (Mielants et al., 1988, 1991, 1995a, 1995b). The aim of our study was to assess whether the eight CD risk loci previously shown to be most strongly associated with both CD and AS (Danoy et al., 2010) were associated with non-invasive markers of gut inflammation in patients with AS, thereby suggesting a potential role for these genes in the aetiology of bowel inflammation. Of the loci identified by Danoy et al. (2010), several have strong functional associations with inflammatory pathways. *JAK2* (rs10758669) and *STAT3* (rs744166) are associated with mucosal immuno-regulation and more widely in immune-mediated disease (O’Shea & Plenge, 2012). Loci associated with cytokines are also likely to be of relevance to mucosal inflammation, particularly IL12 and IL23; indeed, a combined monoclonal antibody therapy targeting these cytokines has proven to be an effective therapy in both IBD and psoriatic arthritis (Feagan et al., 2016; Lebwohl et al., 2015). Other loci identified have less obvious functional association with IBD.

In our study cohort two CD risk loci showed evidence of association with AS, but after Bonferroni correction for multiple testing only the SNP rs11584383 in the chromosome region 1q32 remained significant. The lack of association of the other confirmed CD loci with AS, may be a type II error. Power calculations using the ORs from Danoy et al. (2010), found our cohort had 79.5% power to detect an association with 1q32 with an effect size of 1.35, but only 30–40% power to detect association of the other SNPs with AS.

The minor allele of 1q32 SNP rs11584383 conferred significant protection against AS. This direction of effect was consistent with the direction previously reported in CD. Stratification of the 1q32 SNP rs11584383 genotypes of AS patients, firstly by DISQ score and then by faecal calprotectin had no effect, indicating the association of rs11584383 with AS is independent of non-invasive markers of bowel inflammation. Indeed it is possible that 1q32 SNP rs11584383 is not a marker of bowel inflammation at all, but rather a marker of axial inflammation. The 1q32 SNP rs11584383 was only identified as a risk gene for CD in a meta-analysis of 3,230 CD patients and 4,829 controls. It is estimated that 10–50% of CD patients have sacroiliitis. In this combined cohort the number of CD patients affected by sacroiliitis may have been sufficient for an association of 1q32 SNP rs11584383 with axial inflammation to be detected, but incorrectly attributed to bowel inflammation.

The 1q32 SNP rs11584383 is an intergenic variant (Barrett et al., 2008; Danoy et al., 2010). Therefore, how it may influence inflammation, whether it is axial or bowel-related, is a matter of conjecture. It is possible that 1q32 SNP rs11584383 is in linkage disequilibrium with a functional SNP in a nearby gene. The mostly likely candidate is Kinesin Family Member 21B. This gene is highly expressed in key immune cells, including CD4+ and CD8+ T cells, natural killer cells and B cells. The 1q32 region has also been implicated in AS in Asian populations. In a genome-wide association study investigating copy number variation (CNV) in Korean patients, a deletion type CNV of the 1q32 was found to confer significant risk of developing AS (Jung et al., 2014).

Another possibility is that the 1q32 SNP (rs11584383) is itself functional, and is involved in the regulation of one or more genes. Data from GWAS have shown disease associated intergenic SNPs are often located in or near regulatory elements, suggesting that these variants may to disrupt or alter the function of key regulatory regions of specific genes (1). To determine whether this is the case for the rs115843383 would be challenging (1). However, a number of recently developed bioinformatic techniques, such as Chromosome Conformation Capture (2), Hi-C (3), and chiA-PET (4); which identify long-range chromatin interactions may help to determine whether 1q32 SNP (rs11584383) indeed has a role in the regulation of one or more genes.

There is a wide body of evidence supporting the concept that defective gut mucosal immunity is a factor in initiating and perpetuating spondyloarthritis. The specific combination of genetic variants seems likely to determine whether an individual develops a more spondyloarthritis or IBD phenotype (Brown, Kenna & Wordsworth, 2016). In our study we were unable to demonstrate an association between shared risk genes and clinical features of bowel inflammation in patients with AS.

Our study has several strengths. The study population met strict classification criteria for AS, came from a predominant Northern European genetic population and was carefully phenotyped. In particular, this is the first study to correlate new non-invasive markers of gut inflammation with genetic risk loci, enabling us to look at a much larger population than invasive endoscopic studies would allow.

Potential weaknesses of our study include the small sample size which may have meant that modest associations were missed. However, the stringent phenotyping of our study participants and our decision to only look at loci which had previously been demonstrated to be strongly associated with AS in ethnically comparable populations likely reduced the impact of our small sample size. We chose not to use the DISQ as a continuous variable and this may have reduced our ability to detect a significant correlation with specific loci. We used categorical values to define clinical case definitions of significant bowel symptoms, based on the original validation of the DISQ in AS. Using DISQ scores rather than case defined grouping would be to assign undue significance to subjective self-reported questionnaire values, when compared with categorical objective genetic testing. Such case definitions based on questionnaire values are routinely used in the study of other human diseases, such as depression. Particularly when these are correlated with genetic testing (Taylor et al., 2014).

We did not stratify for ethnicity, although our population was overwhelmingly of Northern European genetic lineage. More recent GWAS in AS have identified further shared genes with SNPs having the same direction of effect in IBD and AS (International Genetics of Ankylosing Spondylitis Consortium (IGAS), 2013). Only around one quarter of the genetic contribution to AS has so far been identified with most of this (approximately 20%) attributable to *HLA-B27* alone, an allele which is not associated with IBD risk (Brown, Kenna & Wordsworth, 2016). Therefore, although we did not demonstrate an association between symptoms and the risk genes we studied, it is possible that other genes alone or in combination may associate with the identified increase in non-invasive markers of bowel involvement.

To our knowledge, this study is the first to stratify confirmed CD risk genotypes with presence of non-invasive makers of bowel inflammation in AS patients, in order to determine whether the association of these genotypes with AS is primarily due to presence of bowel inflammation. Using this approach, we provide preliminary evidence that the association of 1q32 SNP rs11584383 with AS is independent of bowel inflammation as assessed by non-invasive markers. To strengthen and confirm this preliminary finding, further studies which incorporate histology or colonoscopy are needed.

## Supplemental Information

10.7717/peerj.5088/supp-1 Supplemental Information 1 Raw data.

## Additional Information and Declarations

### Data Availability

The following information was supplied regarding data availability: The raw data are provided in a Supplemental File.
